# Health Care–Seeking Behaviors, Disease Progression, Medications, Knowledge of, and Attitudes Toward Systemic Lupus Erythematosus in China: Cross-sectional Survey Study

**DOI:** 10.2196/44541

**Published:** 2023-04-07

**Authors:** Zonglin Dai, Xinxiang Huang, Fei Yuan, Tianwang Li, Baozhao Xie, He Lin, Pingting Yang, Xueyi Li, Shuiming Xu, Jinjun Zhao, Yukai Wang, Xiang Peng, Simin Wei, Wei Huang, Jingyang Li, Jing Liang, Xiuhua Liu, Yongliang Chu, Zhiming Zhang, Renpeng Zhang, Eric H Y Lau, Zhiming Lin

**Affiliations:** 1 School of Public Health, Li Ka Shing Faculty of Medicine, The University of Hong Kong Hong Kong Hong Kong; 2 Department of Rheumatology and Immunology, The People's Hospital of Guangxi Zhuang Autonomous Region Nanning China; 3 Department of Rheumatology, Dongguan People's Hospital Dongguan China; 4 Department of Rheumatology and Immunology, Guangdong Second Provincial General Hospital Guangzhou China; 5 Department of Rheumatology and Immunology, The Seventh Affiliated Hospital, Guangxi Medical University Wuzhou China; 6 Department of Rheumatology and Immunology, Fujian Provincial Hospital, Shengli Clinical Medical College of Fujian Medical University Fujian China; 7 Department of Rheumatology and Immunology, The First Affiliated Hospital of China Medical University Shenyang China; 8 Department of Rheumatology, The Second Affiliated Hospital of Xi'an Jiaotong University Xi'an China; 9 Department of Rheumatology and Immunology, Ganzhou Municipal Hospital Ganzhou China; 10 Department of Rheumatology, Nanfang Hospital, Southern Medical University Guangzhou China; 11 Division of Rheumatology, Shantou Central Hospital Shantou China; 12 Department of Rheumatology and Immunology, The Sixth Affiliated Hospital of Guangzhou Medical University (Qingyuan People's Hospital) Qingyuan China; 13 Division of Rheumatology, Guigang People's Hospital Guigang China; 14 Department of Rheumatology and Immunology, Hainan General Hospital, Hainan Affiliated Hospital of Hainan Medical University Haikou China; 15 Division of Rheumatology, The Affiliated Zhuzhou Hospital of Xiangya Medical College of Central South University Zhuzhou China; 16 Department of Rheumatology, Huadu Affiliated Hospital of Southern Medical University Guangzhou China; 17 Division of Rheumatology, Liuzhou Worker's Hospital, The Fourth Affiliated Hospital of Guangxi Medical University Liuzhou China; 18 Division of Rheumatology, Guangdong Province Hospital of Traditional Chinese Medicine, Zhuhai Branch Zhuhai China; 19 Division of Rheumatology, The Second Affiliated Hospital of Fujian University of Traditional Chinese Medicine Fuzhou China; 20 Division of Rheumatology, The First People's Hospital of Zhaotong Zhaotong China; 21 Laboratory of Data Discovery for Health, Hong Kong Science Park Hong Kong Hong Kong; 22 Department of Rheumatology and Immunology The Third Affiliated Hospital of Sun Yat-sen University Guangzhou China

**Keywords:** systemic lupus erythematosus, health care–seeking behaviors, disease progression, medications, knowledge, attitudes

## Abstract

**Background:**

Systemic lupus erythematosus (SLE) is a systemic autoimmune disease involving multiple organs throughout the body. The health care–seeking behaviors, disease progression of SLE, and patients' knowledge of and attitudes toward SLE have not been characterized in China.

**Objective:**

The aim of this study was to depict the health care–seeking behaviors, disease progression, and medications in patients with SLE and to examine the factors associated with their disease flares, knowledge, and attitudes toward SLE in China.

**Methods:**

We conducted a cross-sectional survey in 27 provinces in China. Descriptive statistical methods were used to depict the demographic characteristics, health care–seeking behaviors, medications, and health status. Multivariable logistic regression models were used to identify the factors associated with disease flares, medication changes, and attitudes toward SLE. An ordinal regression model was used to examine the factors associated with the knowledge of the treatment guidelines.

**Results:**

We recruited 1509 patients with SLE, and 715 had lupus nephritis (LN). Approximately 39.96% (603/1509) of the patients with SLE were primarily diagnosed with LN, and 12.4% (112/906) developed LN (mean time 5.2 years) from non-LN. Patients whose registered permanent residences or workplaces in other cities from the same province and adjacent provinces seeking health care accounted for 66.9% (569/850) and 48.8% (479/981) of the patients with SLE in the provincial capital cities, respectively. Mycophenolate mofetil was the most commonly used immunosuppressive drug in patients without LN (185/794, 23.3%) and patients with LN (307/715, 42.9%). Femoral head necrosis (71/228, 31.1%) and hypertension (99/229, 43.2%) were the most common adverse event (AE) and chronic disease during treatment, respectively. Change of hospitals for medical consultation (odds ratio [OR] 1.90, 95% CI 1.24-2.90) and development of 1 chronic disease (OR 3.60, 95% CI 2.04-6.24) and AE (OR 2.06, 95% CI 1.46-2.92) and more were associated with disease flares. A pregnancy plan (OR 1.58, 95% CI 1.18-2.13) was associated with changes in medication. Only 242 (16.03%) patients with SLE were familiar with the treatment guidelines, and patients with LN tended to be more familiar with the disease (OR 2.20, 95% CI 1.81-2.68). After receiving treatment, 891 (59.04%) patients changed their attitudes toward SLE from fear to acceptance, and patients with college education or higher (OR 2.09, 95% CI 1.10-4.04) were associated with a positive attitude toward SLE.

**Conclusions:**

A large proportion of patients seeking health care in the provincial capital cities of China migrated from other cities. Persistent monitoring of potential AEs and chronic diseases during SLE treatment and managing patients who changed hospitals for medical consultation are essential for controlling disease flares. Patients had insufficient knowledge about SLE treatment guidelines and would benefit from health education to maintain a positive attitude toward SLE.

## Introduction

Systemic lupus erythematosus (SLE) is a systemic autoimmune disease involving multiple organs throughout the body [1]. With continuous improvements in diagnosis and treatment, the 5-year survival rate of patients with SLE has increased significantly to more than 90% in the past 2 decades [2]. In China, different regions have different levels of medical resources, and the provincial capital cities have better medical resources, which attract patients with SLE from other cities in the province or surrounding provinces [3,4]. Patients may also switch hospitals during treatment to seek better health care in China. However, the variation in medical insurance reimbursements and the pursuit of health care in different cities will result in a higher economic burden to patients, including the costs of transportation and time [5,6]. Therefore, it is crucial to study patients’ health care–seeking behaviors to provide a basis and guidance for the allocation of medical resources in China in the future.

Disease flare is a state of increased disease activity and has been reported to occur in more than 20% of the patients within 2 years after remission [7-10]. Disease progression, including disease flares, has become the most common cause of hospitalization for patients with SLE, and the risk of damage accrual has doubled [11,12]. In addition to 30%-60% of patients diagnosed with lupus nephritis (LN), a common SLE complication at the onset, some patients may develop LN during treatment [13-15]. However, only few studies have examined the disease progression of LN in patients with no LN in China. After the diagnosis of SLE, patients are given medications, including hydroxychloroquine, glucocorticoids, and immunosuppressive and biological agents. Later, the accompanying increase of adverse events (AEs) or chronic diseases may potentially impact the change in these medications [16]. Further, medications may become potentially changed if patients with SLE plan to become pregnant [17-19]. A retrospective study in Japan revealed that 9.8% of the patients with SLE had changed their medications, but the factors associated with the medication change were not assessed [20]. Understanding reasons for the change in the medications may improve the clinical management of patients with SLE in China [21].

The updated 2020 Chinese guidelines for the diagnosis and treatment of SLE and the 2019 Chinese guidelines for the diagnosis and treatment of LN (hereafter referred to as the treatment guidelines) are further divided to standardize the diagnosis and treatment standards, which not only provide a basis for the diagnosis and treatment of SLE but also provide a transparent guide for patients with SLE [21,22]. Knowledge about SLE can increase a patient’s adaptation to SLE and its treatment [23]. Insufficient general medical knowledge of the disease would result in doubts about medications, thereby increasing the risk of poor treatment adherence [24]. Nevertheless, there is a dearth of studies focusing on patients’ familiarity with the treatment guidelines, which reflects the patient’s knowledge of the disease to some extent. Socioeconomic and psychosocial factors impact the prognosis of SLE [25]. Negative attitudes, including fear and depression, reduce treatment compliance [26]. A study conducted in Germany showed that negative emotion was associated with 11% reduction in treatment adherence in patients with SLE [27]. A study in China reported that patients with SLE who had anxiety and depression were more likely to have a lower quality of life [28]. Hence, it is essential to explore patients’ attitudes toward SLE from the time of their diagnosis to treatment in China. To sum up, the purposes of our study were to examine the disease progression and health care–seeking behaviors of patients with SLE, depict their medication use, analyze the factors associated with disease flares, and assess the predictors of familiarity with the treatment guidelines and their attitudes toward SLE.

## Methods

### Study Design

The data set in our study was based on an electronic-based questionnaire designed by rheumatologists, epidemiologists, statisticians, and clinicians from multiple centers. The survey was primarily answered by 20 patients and adjusted according to their feedback, and the final version consisted of 4 parts and 51 questions. The first part of the survey was the basic introduction to the study, which required the patient to read the relevant operations carefully and be familiar with the purpose of the study. The second part was about the baseline characteristics of the patients; the third part was about the time of diagnosis, health status, and medications; and the last part was about the knowledge of the treatment guidelines and attitudes toward SLE. The set of questions included fill-in-the-blank, multiple-choice questions with only 1 answer, and multiple-choice questions with several possible answers. To compare the regularity of disease occurrence, we designed the survey by using a large number of time indicators (eg, when were you first diagnosed with SLE and when were you first diagnosed with LN). The detailed contents of the survey are shown in Multimedia Appendix 1.

### Ethics Approval

Ethics approval for this study was obtained from the ethics committee of the Third Affiliated Hospital of Sun Yat-sen University (reference [2021] 02-312-01) on October 19, 2021. All patients signed the informed consent before participating and answering the questions in this study. Information such as the objectives, methods, and expected outcomes from this study was presented in the consent form. The privacy and confidentiality protection description for the participants was provided in the consent form.

### Tool and Data Collection

This survey was designed using a web-based questionnaire tool (WJX), and a quick response code was generated for distribution in hospitals in China. The survey was filled out by patients with SLE who scanned the quick response code under the guidance of the doctors at each visit. The study period was from June 2021 to December 2021, and data were collected from 105 hospitals and 27 provinces. The inclusion criteria were inpatients or outpatients who were diagnosed with SLE and who signed informed consent. Detailed information on the settings is shown in Table S1 of Multimedia Appendix 2.

To ensure the completeness and credibility of the survey, we made each question mandatory to answer. The key questions were asked in a way (eg, from the diagnosis of the disease to treatment, how many chronic diseases have you developed) such that they were verified by the questions in the subsequent sections (eg, the chronic disease(s) you have developed is/are). The survey had to be filled out in the presence of at least one research doctor. If a patient was not familiar with the way of using the tool or did not understand the way of answering in the relevant columns, the doctor would fill it out.

### Definitions

Health care–seeking behavior refers to the seeking of health care in the provincial capital cities and changing of hospitals during treatment (ie, patients sought health care in a hospital different from where they were primarily diagnosed and treated). Disease progression refers to disease flares and the development of AEs and chronic diseases. Disease flares refer to developing LN from non-LN and relapse in all patients with SLE. The Systemic Lupus Erythematosus Disease Activity Index was used to measure relapse in patients without LN if the score increased by at least 4. The reoccurrence of proteinuria or rapid increase of serum creatinine levels was considered as relapse for patients diagnosed with LN [29,30]. Changes in medication meant switching therapy among immunosuppressive drugs and biological agents after the initial treatment. Development of AEs or chronic diseases during treatment referred to the number of AEs or chronic diseases that increased during treatment compared to that in the patients before they were diagnosed with SLE. Patients having a pregnancy plan refers to those with a desire and plan to have children.

Knowledge of the disease was measured by familiarity with the treatment guidelines, which consisted of 3 categories: unfamiliar, less familiar, and familiar. Patients who had not heard of the treatment guidelines were considered unfamiliar. Patients who did not read the treatment guidelines but obtained limited diagnostic and treatment information from other sources were considered less familiar. Patients who read the treatment guidelines in detail and judged their own conditions and guided their medication based on the treatment guidelines were regarded to be familiar with the treatment guidelines. Attitudes toward the disease varied from diagnosis to treatment and were categorized into 4 types: fear throughout treatment (fear →fear), from fear to acceptance (fear →acceptance), from acceptance to fear (acceptance→fear), and acceptance throughout treatment (acceptance→acceptance).

### Statistical Analyses

Descriptive statistical methods were used to depict the demographic and clinical characteristics of patients with no LN and patients with LN. Proportions were calculated and presented in a graphical and tabular form. Progression of SLE, patients’ health behaviors that included self-discontinuation of patients who stopped the treatment prematurely, irregular treatment of patients who had irregular follow-up and medications, knowledge of the treatment guidelines, and attitudes toward the disease were depicted in a Sankey diagram. Multivariable-adjusted logistic regression models were used to explore the factors associated with the development of LN from non-LN, relapse, and changes in medication. An ordinal regression model was adopted to examine the factors associated with the knowledge of the treatment guidelines. One of the key assumptions of the ordinal regression model is the parallel regression assumption, which requires the coefficients in the cumulative binary logistic regression models to be consistent. The Brant test compares the separate fits to binary logistic regression models and hence was suitably used for assessing the parallel regression assumption for ordinal regression [31]. A logistic regression model was used to investigate the factors associated with attitudes toward the disease.

## Results

### Characteristics of the Patients

The data of 1509 patients were collected: 794 (52.62%) patients without LN and 715 (47.38%) patients with LN, with a mean age of 34.9 years and 34.6 years, respectively (Table 1). The mean age of the patients with a primary diagnosis of SLE with non-LN and LN was 29 years and 28.4 years, respectively; 1412 (93.57%) patients with SLE were females, and 616 (40.82%) had a college or higher education. The monthly income of the patients with LN was lower than that of the patients without LN (*P*=.01). Relapse (*P*<.001), changes in medication (*P*<.001), and development of AEs (*P*=.003) and chronic diseases (*P*<.001) in patients with LN were higher than those in patients without LN. Only 242 (16.03%) patients with SLE were familiar with the treatment guidelines, and patients with LN were more familiar with the treatment guidelines than those without LN (*P*<.001); 891 (59.04%) patients with SLE changed their attitudes toward the disease from fear at primary diagnosis to acceptance during the treatment, whereas the proportion of patients’ attitude changing from acceptance to fear was higher in patients with LN (47/715, 6.6%) than in patients without LN (27/794, 3.4%). Of the 1509 patients with SLE, 489 (32.41%) sought health care in a hospital different from the hospital where they were primarily diagnosed and treated, and 719 (47.64%) had a pregnancy plan. The detailed information of the patients is presented in Table 1.

**Figure 1:**
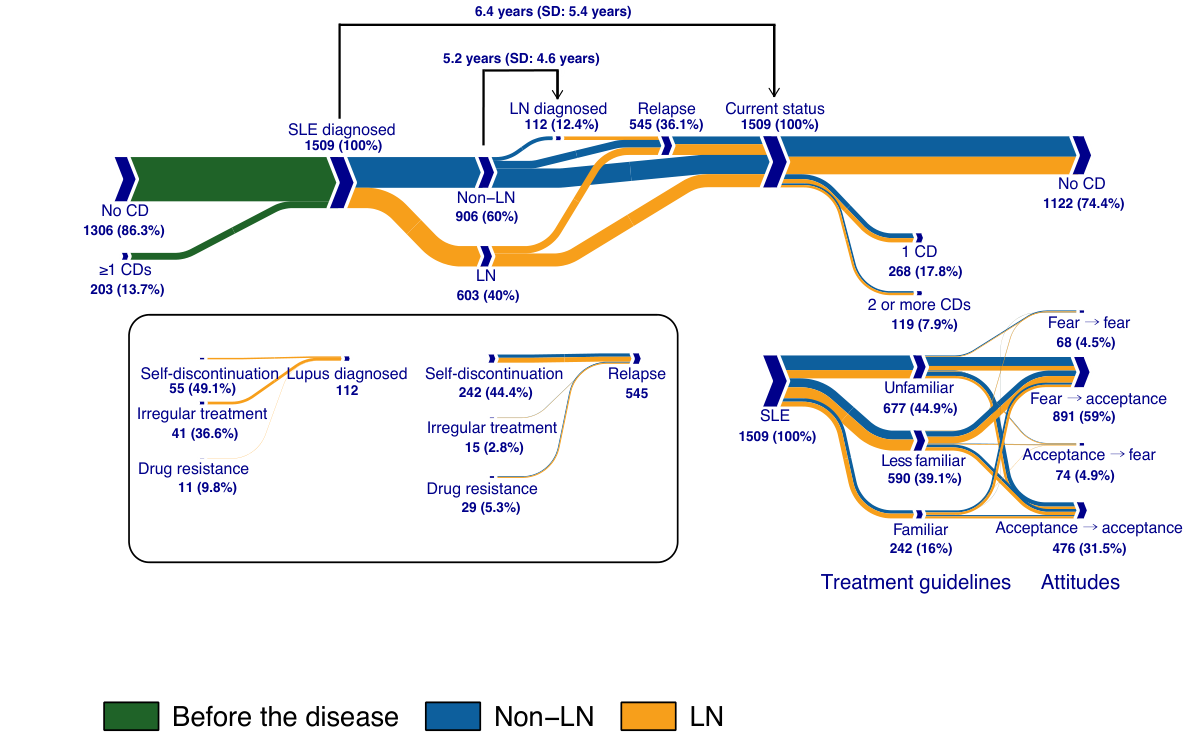
Progression of systemic lupus erythematosus, health behaviors, patients' knowledge of, and attitudes toward the disease. CD: chronic disease; LN: lupus nephritis; SLE: systemic lupus erythematosus.

Figure 1 shows the progression of SLE, health behaviors, and the proportion of patients regarding their familiarity with the disease and attitudes. The mean time for the treatment for patients was 6.4 years. Approximately 39.96% (603/1509) of the patients with SLE were primarily diagnosed with LN, and 12.4% (112/906) of the patients without LN developed LN in the later stage, with a mean time of 5.2 years. Self-discontinuation, irregular treatment, and drug resistance in patients who developed LN from non-LN versus those in patients who experienced relapse were 49.1% (55/112) versus 44.4% (242/545), 36.6% (41/112) versus 2.8% (15/545), and 9.8% (11/112) versus 5.3% (29/545), respectively. Approximately 90.58% (1367/1509) of the patients with SLE developed a positive attitude after treatment.

**Table 1:** Characteristics of the patients with systemic lupus erythematosus (N=1509).

			Patients with no LN^a^ (n=794)		Patients with LN (n=715)		*P* value
Age (years), mean (SD)			34.9 (10.4)		34.6 (10)		.63
Age (years) at diagnosis, mean (SD)			29.0 (10.6)		28.4 (10)		.22
**Gender, n (%)**							.34
	Male	46 (5.8)		51 (7.1)			
	Female	748 (94.2)		664 (92.9)			
**Education, n (%)**							.19
	Junior high school or lower	262 (33)		260 (36.4)			
	Senior high school	191 (24.1)		280 (25.2)			
	College or higher	341 (42.9)		275 (38.5)			
**Monthly income (¥^b^), n (%)**							.01
	<3000	352 (44.3)		372 (52)			
	3000-4999	294 (37)		231 (32.3)			
	≥5000	148 (18.7)		112 (15.7)			
**Basic treatment, n (%)**							
	Hydroxychloroquine	682 (85.9)		596 (83.4)		.20	
	Glucocorticoids	622 (78.3)		589 (82.4)		.06	
**Relapse^c^, n (%)**							<.001
	No	568 (71.5)		396 (55.4)			
	Yes	226 (28.5)		319 (44.6)			
**Changes in medication^d^, n (%)**							<.001
	None	615 (77.5)		464 (64.9)			
	Once	121 (15.2)		152 (21.3)			
	Twice	39 (4.9)		63 (8.8)			
	Three times or more	19 (2.4)		36 (5)			
**Change of hospitals^e^, n (%)**							.60
	No	542 (68.3)		478 (66.9)			
	Yes	252 (31.7)		237 (33.1)			
**Development of adverse events during treatment^f^, n (%)**							.003
	No	694 (87.4)		589 (82.4)			
	One	88 (11.1)		98 (13.7)			
	Two or more	12 (1.5)		28 (3.9)			
**Development of chronic diseases during treatment^f^, n (%)**							<.001
	No	704 (88.7)		576 (80.6)			
	One	65 (8.2)		94 (13.1)			
	Two or more	25 (3.1)		45 (6.3)			
**Pregnancy plan, n (%)**							.77
	No	419 (52.8)		371 (51.9)			
	Yes	375 (47.2)		344 (48.1)			
**Knowledge of the treatment guidelines, n (%)**							<.001
	Unfamiliar	432 (54.4)		245 (34.3)			
	Less familiar	263 (33.1)		327 (45.7)			
	Familiar	99 (12.5)		143 (20)			
**Attitudes toward the disease^g^, n (%)**							.03
	Fear→fear	35 (4.4)		33 (4.6)			
	Fear→acceptance	485 (61.1)		406 (56.8)			
	Acceptance→fear	27 (3.4)		47 (6.6)			
	Acceptance→acceptance	247 (31.1)		229 (32)			

^a^LN: lupus nephritis.

^b^CNY ¥1=US $0.15.

^c^Systemic Lupus Erythematosus Disease Activity Index ≥4 for patients without LN and reoccurrence of proteinuria in patients diagnosed with LN.

^d^Switching therapy among immunosuppressive drugs and biological agents after the initial treatment due to various reasons such as pregnancy plan, drug effectiveness, or serious adverse events.

^e^Patients sought health care in a hospital different from where they were primarily diagnosed and treated.

^f^The development of adverse events or chronic diseases during the treatment compared to that before patients were diagnosed with systemic lupus erythematosus.

^g^The attitude of patients from diagnosis to treatment: fear throughout treatment (fear→fear), from fear to acceptance (fear→acceptance), from acceptance to fear (acceptance→fear), and acceptance throughout treatment (acceptance→acceptance).

### Health Care–Seeking Behaviors

**Figure 2:**
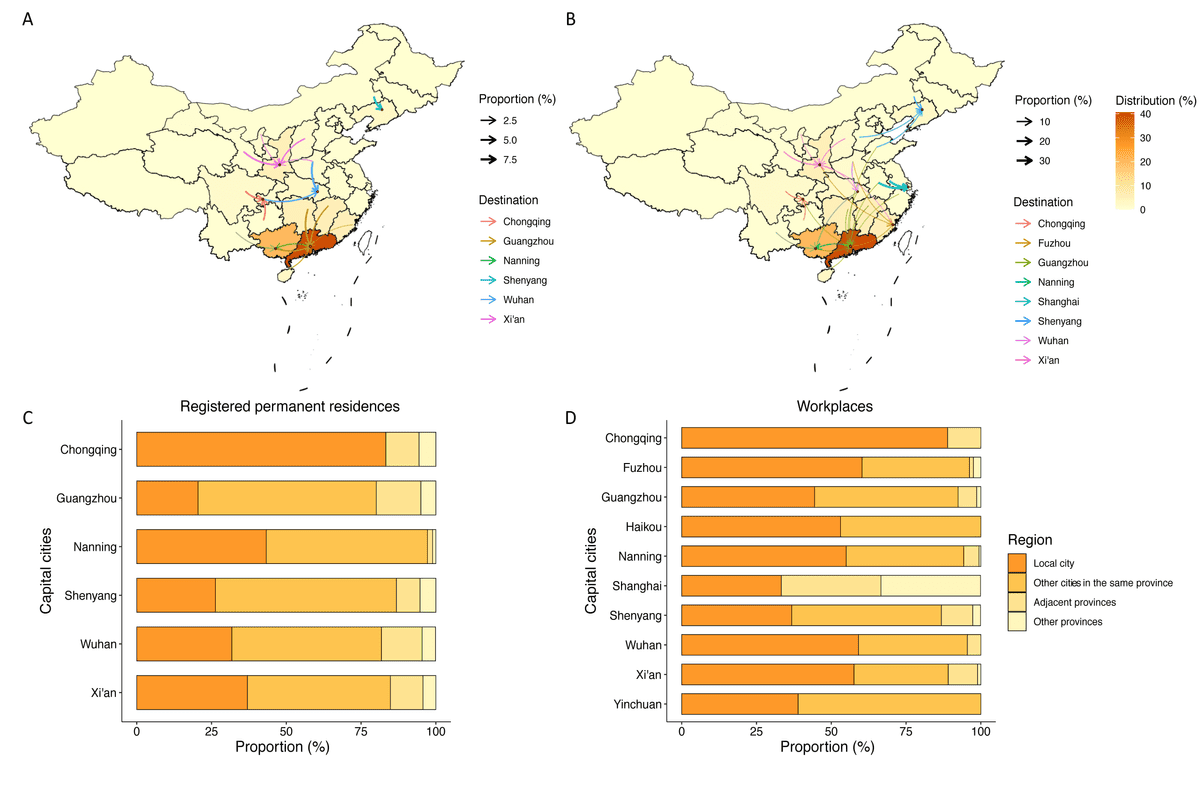
Distribution of the responses of patients with systemic lupus erythematosus in provinces and the health-seeking behaviors in patients from adjacent provinces when considering the registered permanent residences (A) or the workplaces (B), and the proportion of patients from different cities in provincial capital cities when considering the registered permanent residences (C) or the workplaces (D).

The distribution of the responses of patients with SLE and the proportions of patients seeking health care in hospitals in provincial capital cities determined by their registered permanent residence or workplaces are presented in Figure 2. Four provincial capital cities (Wuhan, Nanning, Guangzhou, and Shenyang) attracted over half of the total patients whose registered permanent residences were located in other cities from the same province and the adjacent provinces, ranging from 55.6% (99/178) in Nanning to 74.5% (374/502) in Guangzhou. The proportion reached 66.9% (569/850) in the 6 provincial capital cities. Ten provincial capital cities attracted 48.8% (479/981) of the patients whose workplaces were located in other cities in the same province and from the adjacent provinces, among which Guangzhou and Nanning attracted 54.2% (272/502) and 43.8% (78/178) of the patients, respectively.

### Medications and the Development of AEs and Chronic Diseases

**Figure 3:**
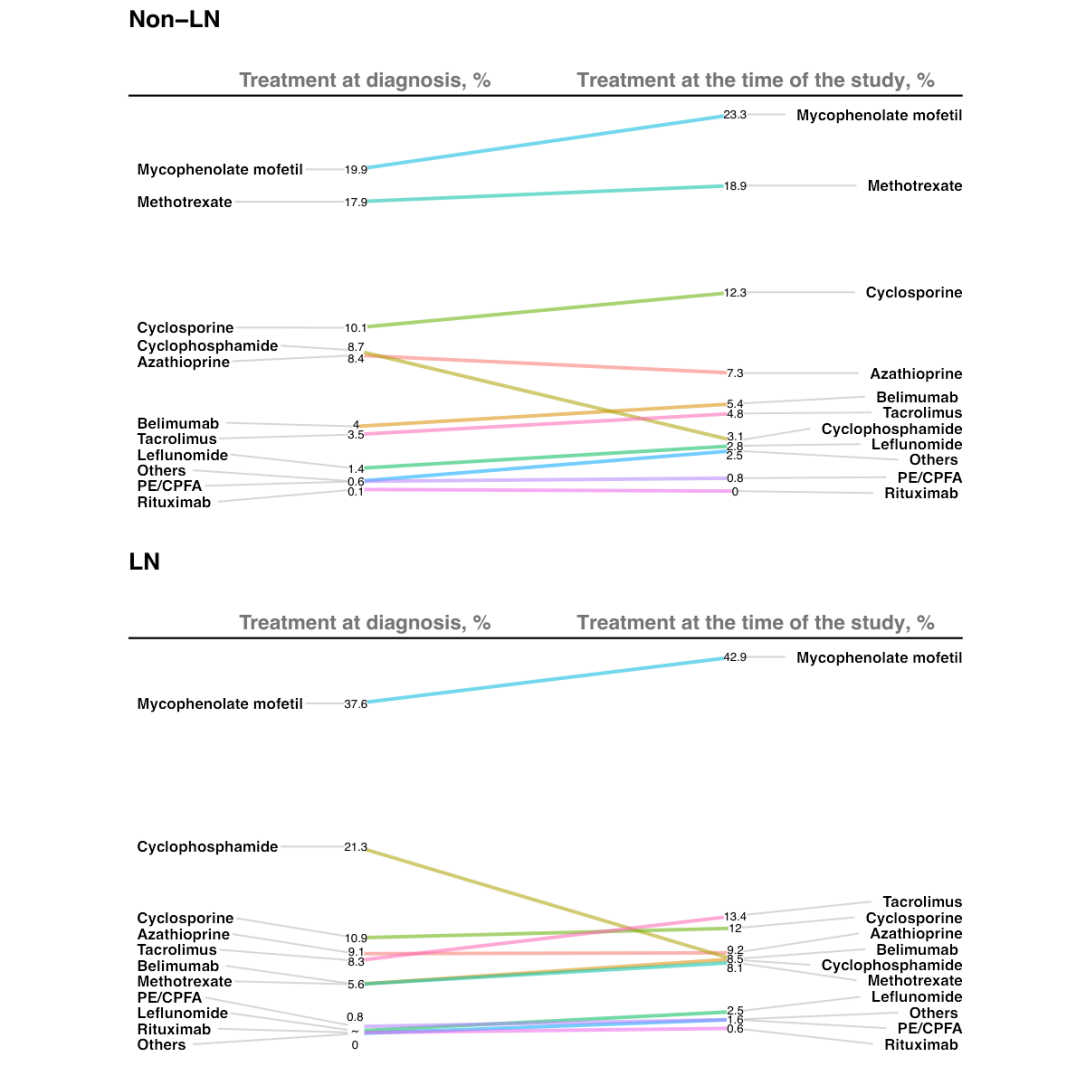
Medication use in patients with systemic lupus erythematosus at diagnosis and treatment during the time of this study. CPFA: continuous plasma filtration absorption; LN: lupus nephritis; PE: plasma exchange.

The most common medication used by patients without LN at the time of the study was mycophenolate mofetil (185/794, 23.3%), followed by methotrexate (150/794, 18.9%) and cyclosporine (98/794, 12.3%). These 3 types of immunosuppressive drugs were also the most commonly used in patients without LN at the time of diagnosis, with 19.9% (158/794) on mycophenolate mofetil, 17.9% (142/794) on methotrexate, and 10.1% (80/794) on cyclosporine, respectively (Figure 3). For patients with LN, the most common medication used at the time of the study was mycophenolate mofetil (307/715, 42.9%), followed by tacrolimus (96/715, 13.4%) and cyclosporine (86/715, 12%). Cyclophosphamide (CYC) (152/715, 21.3%) was the second most commonly used immunosuppressive drug at the time of diagnosis, and only 8.4% (60/715) of the patients used it at the time of the study. Belimumab, one of the biological agents, was used less frequently at the time of the study in patients without LN (43/794, 5.4%) and in patients with LN (61/715, 8.5%).

Concerning the development of AEs and chronic diseases, femoral head necrosis (71/228, 31.1%), cataracts (63/228, 27.6%), and retinal macular degeneration (29/228, 12.7%) were the most common serious AEs that occurred during the treatment, while hypertension (99/229, 43.2%), dyslipidemia (45/229, 19.7%), and chronic gastritis (32/229, 14%) were the 3 common chronic diseases developed during the treatment (Table S2 and Table S3 of Multimedia Appendix 2). The highest educational level (college or higher), change of hospitals, development of AEs and chronic diseases, pregnancy plan, and LN were associated with changes in medication.

### Factors Associated With Disease Flares, Knowledge of, and Attitudes Toward SLE

Change of hospitals (odds ratio [OR] 1.90, 95% CI 1.24-2.90) and development of 1 chronic disease (OR 3.60, 95% CI 2.04-6.24) or 2 or more chronic diseases (OR 4.67, 95% CI 2.13-9.95) were associated with LN development (Table 2). In addition, the development of 1 AE and 2 or more AEs during treatment compared with no AE development and LN status compared with non-LN status was associated with a higher risk of relapse. More factors, namely, age and college or higher education, were significantly associated with medication changes.

Only patients with LN (OR 2.20, 95% CI 1.81-2.68) were associated with familiarity with the knowledge of the treatment guidelines (Table 3). The parallel regression assumption was satisfied by the Brant test (Table S4 of Multimedia Appendix 2). Higher education, including senior high school and college or higher, was associated with a positive attitude to the disease, whereas patients with LN were associated with the risk of a negative attitude, together with the development of 1 AE (table 3).

**Table 2:** Multivariable-adjusted logistic regression analysis of factors associated with disease flares and changes in medication^a^.

			LN development^b^ (n=906), odds ratio (95% CI)		Relapse^c^ (n=1448), odds ratio (95% CI)		Changes in medication^d^ (N=1509), odds ratio (95% CI)
Age			0.98 (0.95-1.00)		1.00 (0.99-1.01)		1.02 (1.00-1.03)^e^
**Gender**							
	Male	Reference		Reference		Reference	
	Female	1.06 (0.47-2.79)		1.00 (0.63-1.62)		1.58 (0.95-2.72)	
**Education**							
	Junior high school or lower	Reference		Reference		Reference	
	Senior high school	1.41 (0.80-2.50)		1.31 (0.97-1.81)		1.33 (0.95-1.86)	
	College or higher	1.28 (0.73-2.27)		1.16 (0.85-1.58)		1.72 (1.24-2.39)^e^	
**Monthly income (¥^f^)**							
	<3000	Reference		Reference		Reference	
	3000-4999	0.71 (0.43-1.15)		0.92 (0.70-1.20)		1.18 (0.89-1.57)	
	≥5000	0.60 (0.30-1.14)		0.75 (0.52-1.08)		1.03 (0.71-1.50)	
**Change of hospitals^g^**							
	No	Reference		Reference		Reference	
	Yes	1.90 (1.24-2.90)^e^		2.23 (1.75-2.84)^e^		1.95 (1.52-2.50)^e^	
**Development of adverse events during treatment^h^**							
	No	Reference		Reference		Reference	
	One	1.73 (0.98-2.97)		2.06 (1.46-2.92)^e^		2.86 (2.04-4.02)^e^	
	Two or more	2.13 (0.73-5.87)		4.29 (1.77-12.06)^e^		4.50 (2.19-9.83)^e^	
**Development of chronic diseases during treatment^h^**							
	None	Reference		Reference		Reference	
	One	3.60 (2.04-6.24)^e^		3.43 (2.34-5.09)^e^		1.90 (1.31-2.75)^e^	
	Two or more	4.67 (2.13-9.95)^e^		2.70 (1.51-4.92)^e^		2.61 (1.50-4.59)^e^	
**Pregnancy plan**							
	No	Reference		Reference		Reference	
	Yes	0.87 (0.52-1.45)		1.01 (0.76-1.34)		1.58 (1.18-2.13)^e^	
**Health status**							
	Non–lupus nephritis	N/A^i^		Reference		Reference	
	Lupus nephritis	N/A		2.03 (1.61-2.56)^e^		1.78 (1.40-2.27)^e^	

^a^All factors were mutually adjusted.

^b^LN: lupus nephritis. The development from non–lupus nephritis to lupus nephritis and the numbers in the analysis included patients with non–lupus nephritis and lupus nephritis in the later stage, excluding patients diagnosed with lupus nephritis.

^c^Systemic Lupus Erythematosus Disease Activity Index ≥4 for patients without lupus nephritis and reoccurrence of proteinuria in patients diagnosed with lupus nephritis. Patients who received treatment without achieving remission were excluded from the analysis.

^d^Switching therapy among immunosuppressive drugs and biological agents after the initial treatment due to various reasons such as pregnancy plan, drug effectiveness, or serious adverse events.

^e^Significant estimates (95% CI).

^f^CNY ¥1=US $0.15.

^g^Patients sought health care in a hospital different from where they were primarily diagnosed and treated.

^h^The development of adverse events or chronic diseases during treatment compared to before patients were diagnosed with systemic lupus erythematosus.

^i^Not applicable.

**Table 3:** Ordinal logistic regression analysis of factors associated with knowledge of the treatment guidelines and logistic regression of factors associated with the attitude toward the disease^a^.

		Knowledge of the treatment guidelines	Attitudes toward the disease^b^	
		Familiar or less familiar versus unfamiliar (N=1509), odds ratio (95% CI)	Fear→acceptance versus fear→fear (n=959), odds ratio (95% CI)	Acceptance→fear versus acceptance→acceptance (n=550), odds ratio (95% CI)
Age		0.99 (0.98-1.00)	1.02 (0.99-1.05)	0.96 (0.93-0.99)^c^
**Gender**				
	Male	Reference	Reference	Reference
	Female	1.33 (0.89-1.99)	0.53 (0.08-1.82)	2.27 (0.76-9.80)
**Education**				
	Junior high school or lower	Reference	Reference	Reference
	Senior high school	1.08 (0.83-1.31)	2.06 (1.05-4.23)^c^	0.63 (0.31-1.22)
	College or higher	1.04 (0.80-1.34)	2.09 (1.10-4.04)^c^	0.71 (0.35-1.40)
**Monthly income (¥^d^)**				
	<3000	Reference	Reference	Reference
	3000-4999	1.19 (0.95-1.50)	1.43 (0.80-2.63)	0.75 (0.40-1.36)
	≥5000	1.28 (0.94-1.73)	2.23 (0.90-6.37)	0.75 (0.28-1.80)
**Change of hospitals^e^**				
	No	Reference	Reference	Reference
	Yes	0.92 (0.74-1.13)	1.15 (0.68-2.00)	0.99 (0.53-1.76)
**Development of adverse events during treatment^f^**				
	No	Reference	Reference	Reference
	One	0.83 (0.61-1.12)	0.93 (0.45-2.13)	2.73 (1.36-5.35)^c^
	Two or more	0.80 (0.42-1.52)	0.37 (0.13-1.26)	0.94 (0.05-5.93)
**Development of chronic diseases during treatment^f^**				
	None	Reference	Reference	Reference
	One	0.95 (0.69-1.32)	0.66 (0.32-1.44)	1.10 (0.45-2.44)
	Two or more	1.25 (0.77-2.02)	0.77 (0.25-2.96)	1.92 (0.58-5.48)
**Pregnancy plan**				
	No	Reference	Reference	Reference
	Yes	0.79 (0.63-1.00)	1.21 (0.65-2.27)	1.76 (0.95-3.28)
**Health status**				
	Non-LN^g^	Reference	Reference	Reference
	LN	2.20 (1.81-2.68)^c^	1.07 (0.64-1.80)	1.86 (1.11-3.16)^c^

^a^All factors were mutually adjusted.

^b^Attitudes of patients from diagnosis to treatment: fear throughout treatment (fear→fear), from fear to acceptance (fear→acceptance), from acceptance to fear (acceptance→fear), and acceptance throughout treatment (acceptance→acceptance).

^c^Significant estimates (95% CI).

^d^CNY ¥1=US $0.15.

^e^Patients sought health care in a hospital different from where they were primarily diagnosed and treated.

^f^The development of adverse events or chronic diseases during the treatment compared to that before patients were diagnosed with systemic lupus erythematosus.

^g^LN: lupus nephritis.

## Discussion

### Principal Results

A noteworthy finding in our study is that 55.6% (473/850) of the patients with SLE who sought health care in the provincial capital cities migrated from other cities in the same province when considering their registered permanent residences. When considering the workplace, hospitals in provincial capital cities also lured 42.9% (421/981) of the patients with SLE from the same provinces to seek health care even if the workplaces of these patients were not located in the capital city. In addition, compared with general provincial capitals, high-income provincial capitals also attracted patients from adjacent provinces such as Guangzhou and Xi’an. This can be partly explained by the push-pull theory, as the provincial capital city has more resources in the province, including more hospital options, more medical experts, and better medical technology, which could be considered as the social factors for patients to achieve better quality of life [32]. However, out-of-town visits will increase patients’ economic burdens such as transportation and accommodation costs. Therefore, targeted measures should be adopted by various departments for these patients. Hospitals in provincial capital cities can establish a unique medical channel that can provide personal support for people who migrate from other cities to seek medical care through effective identification. The medical insurance departments of each province should simplify the medical reimbursement process for patients from other cities in the same province and the adjacent provinces to reduce the economic burden of patients.

Factors associated with relapse in patients with SLE have been reported in many studies, including the increased anti-double-stranded deoxyribonucleic acid, B lymphocyte stimulator, renal organ involvement, deficiency of hydroxychloroquine, premature discontinuation, and poor compliance [22,33-35]. Consistent with the factors above, we found that patients with LN had a higher risk associated with relapse, among which 49.1% (55/112) of the patients self-discontinued the treatment. In addition, our results showed that the development of 1 or more AEs and chronic diseases during treatment increased the risk of disease flares, including the development of LN from non-LN and relapse in all patients with SLE. The mean time of disease progression in Chinese cohorts was 5.2 years, corresponding to an international inception cohort study with a mean time of 4.6 years [14]. The outcomes in our study may extend the previous finding [14].

A novel finding in our study is that switching from the first hospital where patients were primarily diagnosed and treated was associated with their risk of development of LN and relapse and was significantly associated with changes in their medications. This finding implies that the hospital that the patient first visited may have provided improper treatment. Requesting more meticulous care and treatment for patients with a record of switching hospitals from other cities, especially in the provincial capital cities, is imperative because the major public hospitals have a large number of patients from other cities, with outpatient visits and hospitalizations increasing. A study in 1 hospital in Kunming city, a provincial city in the Yunnan province, showed that outpatient visits and hospitalizations increased from 188,530 and 6620 in 2001 to 369,510 and 12,380 in 2010, respectively [36]. Another hospital in Shanghai showed that outpatient visits increased from 734,923 in 2013 to 881,376 in 2017 [37]. Although medical resources are more concentrated in provincial capital cities, individualized management of patients in those cities, including consideration of the patient’s diagnosis and treatment history, at the initial hospital and the patient’s home city is indispensable [3,4].

Apart from factors such as older age, higher education, development of at least 1 AE and chronic disease, change of hospitals, and LN development that were associated with changes in medication, nearly half of the patients with SLE had a pregnancy plan, which was also a significant factor (OR 1.58, 95% CI 1.18-2.13). It has been confirmed that using CYC leads to ovarian failure [38]. Our study shows that the use of CYC decreased by 60.5% (92/152) in the current treatment (at the time of the study) compared with that during the primary treatment, and the proportion of patients using CYC among those with a pregnancy plan was significantly lower than that among those without a pregnancy plan (Table S5 of Multimedia Appendix 2). This finding shows that in addition to considering clinical symptoms, the plans and the needs of the patients are also worth considering by doctors in the treatment process.

The familiarity with treatment guidelines reflects a patient’s knowledge of the disease to a certain extent [39]. Our results indicated that only 12.5% (99/794) of the patients without LN and 20% (143/715) of the patients with LN were familiar with the treatment guidelines. Moreover, only patients with LN were more familiar with the treatment guidelines than patients without LN. The limited proportion of patients with knowledge of the treatment guidelines in China can be worrisome, as these results revealed 2 issues. First, the knowledge of the disease was inadequate; therefore, the treatment decision was more passive or physician-led. Furthermore, the lack of education and knowledge of SLE would increase the risk of disease flares [24]. Second, patients were more familiar with the disease in a severer state than when the disease was in a mild stage, indicating that patients focused less on their disease in the early stage. A study has shown that patients’ knowledge of the disease could be improved by reading the treatment guide [39]. Therefore, the potential solution is to condense and simplify the relevant content of diagnosis and treatment in the treatment guidelines and to distribute the guidelines to doctors and patients in electronic or paper versions in a simple booklet for their study and reference. In addition, patients should be rendered a comprehensive understanding of the disease when the disease status is mild rather than after the disease has worsened.

Patients’ attitudes toward SLE, on the whole, reflected acceptance, with approximately 90.58% (1367/1509) of the patients expressing a positive attitude during the treatment period, although nearly half of the patients felt fear at the time of diagnosis. An important factor was patients with LN who had a higher risk of developing a fearful attitude at diagnosis than patients without LN. One way to improve the patient’s attitude is by providing health education. Studies have indicated that good education is associated with higher knowledge of diseases and increased self-awareness of one’s health and assessment of health care [40-42]. Further, the interaction between doctors and patients can be strengthened, including using the evaluation function of the survey (WJX) to test patients’ understanding of the latest research progress on the disease and monitoring patients’ attitude changes to the disease at different stages, which can be achieved through a series of scoring and incentive measures [43].

### Limitations In This Study

Our findings have some limitations and must be interpreted with caution. First, although we set the time points for collecting variables, the chronological order of the factors and the outcomes might not be explicit. For example, we dynamically described a change in the development of chronic diseases before and after the diagnosis of SLE. However, whether its increase occurs before or after the development of LN or relapse has not been elucidated. This limitation is because, on the one hand, the study design was cross-sectional. On the other hand, even in the existing medical database, the corresponding time point is relatively vague; therefore, reverse causality or simultaneity may appear. Second, a more detailed measurement of health emotions may improve health decision-making [44-46]. Our study lacked such measurement of attitudes toward SLE, and a further study evaluating patients’ emotions on a numerical scale may improve the findings’ accuracy. Third, our study might have responder bias. Since Guangdong is the most populous province in China, more samples were collected from Guangdong, while samples from other regions were more evenly distributed. Furthermore, recall bias might arise in the data collection process, especially for those who had received long-term treatment for more than 10 years. It was challenging for patients to recall their physical condition and medication regimen before the diagnosis. In order to reduce this limitation, we set the number of entries and specific names in the survey to achieve consistency and to ensure that recall bias was minimized.

### Conclusions

In our study, a large proportion of patients seeking health care in provincial capital cities migrated from other cities in the same province and the adjacent provinces. Mycophenolate mofetil was the most commonly used immunosuppressive drug in the treatment of patients without LN and patients with LN at the time of this study. Femoral head necrosis was the most common AE, followed by cataract and retinal macular degeneration, while hypertension, dyslipidemia, and chronic gastritis were the 3 common chronic diseases developed during the treatment. Change of the hospital where patients were primarily diagnosed and treated and the development of AEs and chronic diseases were associated with disease flares and changes in medication. A pregnancy plan was also associated with changes in medication. Patients had insufficient knowledge about the treatment guidelines, and patients without LN tended to be less familiar with the treatment guidelines compared to patients with LN. Patients with SLE who had college education or higher were associated with a positive attitude rather than those who had only a junior high school education or lower. Our findings suggest that disease flares should be controlled by monitoring potential AEs and chronic diseases during treatment and by effectively managing patients who switched hospitals for their medical consultation. In addition, patients would benefit from health education to maintain a positive attitude toward SLE.

## Appendix

Multimedia Appendix 1 Survey used in this study.

Multimedia Appendix 2 Supplementary Tables 1-5. Table S1 shows the settings of patients with systemic lupus erythematosus from registered permanent residences or workplaces in mainland China. Table S2 and Table S3 present the number of adverse events and chronic diseases developed in patients with systemic lupus erythematosus during the treatment, respectively. Brant test for each parameter regarding the parallel regression assumption in an ordinal logistic regression model is reflected in Table S4, and Table S5 is the use of medications in patients with systemic lupus erythematosus regarding the pregnancy plan at the time of the study.

## Abbreviations

AE: adverse event
CYC: cyclophosphamide
LN: lupus nephritis
OR: odds ratio
SLE: systemic lupus erythematosus

## Data Availability

The data are not publicly available due to the potential violation that may compromise the privacy of the research participants. However, data may be available upon proper request from the corresponding author.
