# Supplementary material for: Health Care–Seeking Behaviors, Disease Progression, Medications, Knowledge of, and Attitudes Toward Systemic Lupus Erythematosus in China: Cross-sectional Survey Study
Source: JMIR Public Health Surveill. 2023 Apr 7;9:e44541. doi: 10.2196/44541 (PMC10131714; doi:10.2196/44541)
Supplement: Multimedia Appendix 2 [file publichealth_v9i1e44541_app2.docx]

**Table S1.** Settings of patients with systemic lupus erythematosus from registered permanent residences or workplaces in mainland China.

| Districts | From registered permanent residences | | From workplaces | |
| --- | --- | --- | --- | --- |
|  | Number | % | Number | % |
| Hebei | 3 | 0.2 | 2 | 0.1 |
| Shanxi | 21 | 1.4 | 18 | 1.2 |
| Inner Mongolia | 4 | 0.3 | 3 | 0.2 |
| Liaoning | 35 | 2.3 | 34 | 2.3 |
| Jilin | 2 | 0.1 | 0 | 0 |
| Heilongjiang | 2 | 0.1 | 0 | 0 |
| Shanghai | 1 | 0.1 | 1 | 0.1 |
| Jiangsu | 10 | 0.7 | 13 | 0.9 |
| Zhejiang | 3 | 0.2 | 4 | 0.3 |
| Anhui | 10 | 0.7 | 3 | 0.2 |
| Fujian | 85 | 5.6 | 83 | 5.5 |
| Jiangxi | 83 | 5.5 | 63 | 4.2 |
| Shandong | 3 | 0.2 | 5 | 0.3 |
| Henan | 13 | 0.9 | 2 | 0.1 |
| Hubei | 37 | 2.5 | 27 | 1.8 |
| Hunan | 75 | 5 | 26 | 1.7 |
| Guangdong | 613 | 40.6 | 775 | 51.4 |
| Guangxi | 307 | 20.3 | 274 | 18.2 |
| Hainan | 44 | 2.9 | 42 | 2.8 |
| Chongqing | 19 | 1.3 | 17 | 1.1 |
| Sichuan | 22 | 1.5 | 1 | 0.1 |
| Guizhou | 3 | 0.2 | 2 | 0.1 |
| Yunnan | 5 | 0.3 | 3 | 0.2 |
| Shaanxi | 82 | 5.4 | 85 | 5.6 |
| Gansu | 7 | 0.5 | 7 | 0.5 |
| Ningxia | 19 | 1.3 | 19 | 1.3 |
| Xinjiang | 1 | 0.1 | 0 | 0 |

**Table S2.** The number of adverse events developed in patients with systemic lupus erythematosus during the treatment^a^.

| Adverse events | Cases^b^ | Proportion (%) |
| --- | --- | --- |
| Femoral head necrosis | 71 | 31.1 |
| Cataract | 63 | 27.6 |
| Retinal macular degeneration | 29 | 12.7 |
| Serious infection | 26 | 11.4 |
| Other ocular diseases^c^ | 19 | 8.3 |
| Glaucoma | 17 | 7.5 |
| Osteopenia | 10 | 4.4 |
| Alopecia | 8 | 3.5 |
| Gynecological disease | 7 | 3.1 |
| Shingles | 6 | 2.6 |
| Mild infection | 5 | 2.2 |
| Activity limitation | 4 | 1.8 |
| Liver lesion | 2 | 0.9 |
| Thrombus | 1 | 0.4 |
| Facial numbness | 1 | 0.4 |
| Hip joint effusion | 1 | 0.4 |
| Septicemia | 1 | 0.4 |

^a^The total number of patients who developed one or more adverse events during the treatment was 228.

^b^The number of adverse events developed in patients when they received treatment.

^c^Ocular diseases included retinal macular degeneration, cataract, glaucoma, and other ocular diseases.

**Table S3.** The number of chronic diseases developed in patients with systemic lupus erythematosus after diagnosis and received treatment^a^.

| Adverse events | Cases^b^ | Proportion (%) |
| --- | --- | --- |
| Hypertension | 99 | 43.2 |
| Dyslipidemia | 45 | 19.7 |
| Chronic gastritis | 32 | 14 |
| Chronic lung disease | 23 | 10 |
| Cardiovascular disease | 21 | 9.2 |
| Diabetes | 16 | 7 |
| Liver disease | 14 | 6.1 |
| Arthritis or rheumatism | 14 | 6.1 |
| Tumor | 12 | 5.2 |
| Chronic bronchitis | 12 | 5.2 |
| Thyroid disease | 9 | 3.9 |
| Stroke | 7 | 3.1 |
| Sjogren syndrome | 3 | 1.3 |
| Vasculitis | 3 | 1.3 |
| Osteoporosis | 3 | 1.3 |
| Chronic skin disease | 2 | 0.9 |
| Renal disease | 2 | 0.9 |
| Neuropsychiatric lupus | 2 | 0.9 |
| Myelitis | 2 | 0.9 |
| Scleroderma | 2 | 0.9 |
| Chronic pancreatitis | 2 | 0.9 |
| Urethritis | 1 | 0.4 |
| Osteoproliferation | 1 | 0.4 |
| Benign prostatic hyperplasia | 1 | 0.4 |
| Cyclomastopathy | 1 | 0.4 |
| Chronic thrombocytopenic purpura | 1 | 0.4 |
| Chronic cholecystitis | 1 | 0.4 |
| Depression | 1 | 0.4 |
| Chronic pharyngitis | 1 | 0.4 |

^a^Systemic lupus erythematosus and lupus nephritis were excluded. The total number of patients who developed one or more adverse events during the treatment was 229.

^b^The number of adverse events developed in patients when they received treatment.

**Table S4.** Brant test for each parameter regarding the parallel regression assumption in an ordinal logistic regression model.

| Parameters | *P* value |
| --- | --- |
| Age | .78 |
| **Gender** |  |
| Male | Reference |
| Female | .48 |
| **Education** |  |
| Junior high school or lower | Reference |
| Senior high school | .32 |
| College or higher | .91 |
| **Monthly income (¥^a^)** |  |
| <3000 | Reference |
| 3000-4999 | .33 |
| ≥5000 | .42 |
| **Change of hospitals^b^** |  |
| No | Reference |
| Yes | .08 |
| **Development of adverse events during treatment^c^** |  |
| None | Reference |
| One | .73 |
| Two or more | .07 |
| **Development of chronic diseases during treatment^c^** |  |
| None | Reference |
| One | .71 |
| Two or more | .63 |
| **Pregnancy plan** |  |
| No | Reference |
| Yes | .74 |
| **Health status** |  |
| Non-LN^d^ | Reference |
| LN | .06 |

^a^CNY ¥1=US $0.15.

^b^Patients sought healthcare in a hospital other than the hospital where patients were primarily diagnosed and treated.

^c^The development of adverse events or chronic diseases during the treatment was compared to before patients were diagnosed with systemic lupus erythematosus.

^d^LN, lupus nephritis.

**Table S5.** The use of medications in patients with systemic lupus erythematosus regarding the pregnancy plan at the time of the study.

| Medications | With a pregnancy plan (n=719) | Without a pregnancy plan (n=790) | *P* value |
| --- | --- | --- | --- |
| Cyclophosphamide, n (%) | 29 (4) | 56 (7.1) | .01 |
| Mycophenolate mofetil, n (%) | 256 (35.6) | 236 (29.9) | .02 |
| Azathioprine, n (%) | 63 (8.8) | 61 (7.7) | .52 |
| Rituximab, n (%) | 2 (0.3) | 2 (0.3) | .91 |
| Tacrolimus, n (%) | 75 (10.4) | 59 (7.5) | .053 |
| Cyclosporine, n (%) | 108 (15) | 76 (9.6) | .002 |
| Methotrexate, n (%) | 84 (11.7) | 124 (15.7) | .03 |
| Belimumab, n (%) | 56 (7.8) | 48 (6.1) | .23 |
| Plasma exchange and/or continuous plasma, n (%) | 10 (1.4) | 7 (0.9) | .49 |
| Leflunomide, n (%) | 14 (1.9) | 26 (3.3) | .14 |
| Others, n (%) | 13 (1.8) | 19 (2.4) | .53 |
